# A History of Pain Studies and Changing Attitudes to the Welfare of Crustaceans

**DOI:** 10.3390/ani15030445

**Published:** 2025-02-06

**Authors:** Robert William Elwood

**Affiliations:** School of Biological Sciences, Queen’s University, Belfast BT9 5DL, UK; r.elwood@qub.ac.uk

**Keywords:** decapod, humane slaughter, welfare, sentience

## Abstract

Early discussions about possible pain in crustaceans often comprised speculation and anecdotes. Experiments to investigate pain took guidance from these early debates, and from studies on other taxa. Many experiments provided data that are consistent with the idea of pain. However, that does not mean that pain has been proved, but they open the possibility. With each study that is consistent with pain being felt, the probability increases, but we will probably never have conclusive proof. Some responses appear to be nociceptive reflexes; however, they at least indicate that the animal responds to stimuli such as tissue damage, heat, acid, alkaline, or electric shock. The data are said to be consistent with pain only if they cannot be explained by reflexes. These studies have encouraged various organisations to improve the welfare of crustaceans, e.g., PETA, Crustacean Compassion, RSPCA, British Veterinary Association, UFAW, and HSA. They also formed much of the evidence included in the highly influential review of sentience for the UK government that resulted in the inclusion of decapod crustaceans in the Animal Welfare (Sentience) Act 2022. This recognises that decapod crustaceans are sentient. By contrast, the New York Declaration recognises there is a possibility of sentience. Others have argued against the idea of pain in crustaceans. Nevertheless, the 2022 Act provided an impetus to groups that campaign for improved welfare. Some UK retailers now require improved slaughter techniques for the shrimp/prawn they sell, and electrical stunning is the preferred slaughter method.

## Simple Summary

We cannot know if crustaceans feel pain, but recent experimental studies suggest that it is a possibility. Crustacean responses to noxious stimuli show many of the traits seen in vertebrates. This has led to changes in the way these animals are perceived, and several key animal welfare groups have pushed for their protection. They have been recognised recently as being sentient in UK law, but that has not changed the legality of various treatments. There is, however, a consideration in progress about their use in science but not commercial practises. Nevertheless, major retailers have made demands of their suppliers to alter how these animals are treated in rearing and processing, including slaughter, and there has been research into methods that might increase the welfare of the billions of these animals consumed by humans each year.

## 1. Introduction

Various crustaceans, particularly decapods, are consumed by humans [1,2]. These include lobsters, crawfish, crayfish, crabs, prawns, and shrimp, which are ether wild-caught or farmed, and the numbers are vast. In 2023, Waldhorn and Autric [3] estimated that between 7.6 and 76 trillion (mean 27 trillion) shrimp and prawns (these terms are used interchangeably and have no taxonomic meaning) are killed each year. They note this is about 255 times the number of farmed fishes killed per year, and about 18 times the current estimates of fishes captured from the wild [3].

Decapods are often treated in extreme ways that would be illegal for cattle or poultry. Most are killed by suffocation, chilling, or by boiling [2]. However, some are killed by “butchering” or “mutilation”. For example, in some crab fisheries, the claws are pulled and/or twisted from the main body, and the still live animal is then thrown back to the sea to die [4,5]. Other examples include lobsters having their claws pulled off during processing, then having the walking legs cut off, and then the animal being impaled on a spike so the abdominal (tail) meat can be extracted [6]. The lobster typically remains alive during these actions, and takes minutes to die. There is no attempt to stun or kill the animal [3]. Further, in aquaculture systems, prawns have their eyestalks ablated so that the subsequent hormonal changes encourage egg production and thus enhance reproduction [7]. Live crustaceans may also be held in tanks and transported for days, with claws either bound or mutilated so that they are non-functional [8]. Crustaceans are also used in science, and there is little regulation in the UK or elsewhere about what may be done to the animals [9]. Until relatively recently, these treatments were not questioned and there was little interest in the potential for pain (a negative emotional state) or suffering within this group [10].

## 2. Early Thoughts on Possible Pain

Fiorito (1986) [11] briefly reviewed largely anecdotal evidence about possible pain and concluded that, for invertebrates, “*The evidence available until now from the neural organization and behaviour patterns does not allow a conclusive picture about the existence of a pain-like system in these animals. However, the role of opioid peptides in the regulation of nociception, in the control of reactions produced by painful stimuli, and in procuring analgesia as well as behavioural responses (such as escape, defence, mobbing, avoidance) to it, makes it possible to conclude that some pain system has appeared in invertebrates*”. Whilst this seems to be sitting on the fence, or even contradictory, there is a consideration that some invertebrates might experience pain. However, the idea of describing stimuli as painful and using those stimuli to infer pain is a circular argument. Eisemann et al. (1984) [12] also discussed anecdotal evidence and experiments, some of which did not directly address the question of pain. He concluded that pain was highly unlikely in invertebrates, with the possible exception of cephalopods. However, he made it clear that a conclusive answer could not be provided because the subjective experience of any organism cannot be experienced by another.

These reviews and considerations rested on the argument by analogy. That is, if an invertebrate was seen to respond to a stimulus that would cause pain to a human in a similar way that seen in humans, then we may suspect that the response was mediated in ways like those in humans. This argument was developed by Sherwin (2001) [13] in reviewing several aspects of responses to noxious stimuli. These aspects included memory, learning, cognitive abilities, protective responses and autotomy, and physiological changes. Invertebrate examples of each were noted to have similarities, at least in part, to those of vertebrates and, thus, could be used in arguments by analogy. However, he noted there was a reluctance to use the argument with respect to invertebrates, possibly because of the inconvenience to humans by accepting that pain may be experienced by these animals. Sherwin (2001) [13], however, noted that to apply the key argument to vertebrates but not to invertebrates was illogical, but he did not make a strong claim for pain in invertebrates. He stressed the need for further research, and wrote: “*Precious little work has been directed to specifically investigating whether the widely perceived dichotomy between vertebrates and invertebrates with their experiencing negative mental states has a justifiable basis*”.

David Foster Wallace wrote about the Maine Lobster Festival in 2004 [14] for the Gourmet magazine. Whilst the well-known article, “*Consider the lobster*”, was not an academic paper, it was both thoughtful and influential about the possibility of pain. He discussed the reasons for and against the idea of pain, and ways that it might be minimised by killing lobsters prior to boiling. His conclusion about their responses to boiling was “*it is hard to deny in any meaningful way that this is a living creature experiencing pain and wishing to avoid/escape the painful experience*”.

Further, Broom (2007) [15] wrote: “*There is evidence from some species of fish, cephalopods and decapod crustaceans of substantial perceptual ability, pain and adrenal systems, emotional responses, long- and short-term memory, complex cognition, individual differences, deception, tool use, and social learning. The case for protecting these animals would appear to be substantial*”. That is, by 2007, there had been several publications that either supported the idea of sentience (the ability to have feelings) in crustaceans or said that sentience was a possibility.

## 3. Experiments on the Possibility of Pain in Crustaceans

### 3.1. Directed Responses Following Application of Noxious Stimuli

The first empirical study that asked if pain was possible in decapods examined responses of glass prawns (*Palaemon elegans*) following noxious stimuli was Barr et al. (2008) [16]. It also examined if pretreatment with a local anaesthetic would reduce activities that might be pain-related. Animals were first removed from the water, and one antenna was brushed with either water or benzocaine and returned to the water. After observing for 5 min, the animals were again removed and the same antenna was treated with acetic acid, sodium hydroxide, water, or pinched with forceps, and then returned to the water. Animals first treated with seawater were more likely to show a tail-flick escape response when a noxious treatment was applied than were those first treated with benzocaine, indicating an expected effect of local anaesthesia. Further, animals treated with the noxious chemicals after pretreatment with water rubbed the treated antenna against the side of the tank and groomed the treated antenna by pulling it through the small claws on the anterior walking legs. Both activities were reduced by prior benzocaine treatment. Pinching also resulted in increased rubbing of the antenna (but not grooming), but this activity was not reduced by prior benzocaine treatment. Tail-flicking was regarded as consistent with a nociceptive reflex, but the prolonged rubbing and grooming activities directed at the specific antenna, which were both reduced by the local anaesthetic, were regarded as “*consistent with the idea that these crustaceans can experience pain*”.

A subsequent study by Puri and Faulkes (2010) [17], however, found markedly different results. It was said to replicate the study of Barr et al. [16] with three other species of decapod crustaceans: white shrimp (*Litopenaeus setiferus*), grass shrimp (*Palaemonetes* sp.), and Louisiana red swamp crayfish (*Procambarus clarkii*). One antenna of each was brushed with either benzocaine dissolved in ethanol or just ethanol, or with sodium hydroxide, hydrochloric acid, or water. No evidence of increased directed grooming or rubbing of the antennae was reported. By contrast, Dyuizen (2012) [18] reported that injection of formalin into the claw of a shore crab, *Hemigrapsus sanguineus*, resulted in marked behavioural changes, including reduced use of that appendage during locomotion, increased rubbing of the claw with the alternative claw and walking legs, holding the injected claw against the carapace, and 20% of those in the formalin group autotomised the claw, compared to 0% in the control group [18].

Several further studies reported distinct responses to noxious chemicals. These include marked behavioural responses to acetic acid on the eyes or mouthparts of the shore crab, *Carcinus maenas* [19], complex and prolonged rubbing of the eyes by glass prawns, *P. elegans*, when they were brushed with sodium hydroxide [20], and rubbing, accompanied by changed use of rear walking legs, of shore crabs when injected with acetic acid [21]. Other examples of activities directed towards the site of a noxious stimulus include grooming the abdomen following shocks to that location in hermit crabs [22] and brown crabs, *Cancer pagurus*, that guarded their wound following claw removal [23].

The negative result of Puri and Faulkes [17] appears to be an outlier, and are not easily explained. Puri and Faulkes [17] used three species, and found similar results for all of them. However, the sample sizes, experimental design, and analyses appear to differ to those of Barr et al. (2008) [16]. Barr et al. [16] used a two-stage design with application of water or benzocaine to one antenna in the first stage and then water, NaOH, acetic acid, or pinching to the same antenna in the second. The sample size was 144, or 288 when activities directed at each of the paired antennae were examined. Analyses used statistically powerful three-way ANOVAs with repeated measures (treated and untreated antennae), which allows for the interactions between factors to be explored. By contrast, Puri and Faulkes [17] did not use a two-stage design, and write: “*Half the individuals swabbed with a control (water or sea water for NaCl and HCl; ethanol for benzocaine) on the distal half of one second antenna, and half were swabbed the stimulus. Thus, each individual had an antenna that was not swabbed, so that any effects of the mechanical action of swabbing alone could be detected*”. The sample size was 17 to 24 for each analysis. That is, even the largest sample was less than 10% of that used by Barr et al. [16]. Independent t tests were used, although treated and untreated antennae were compared, which would have required paired t tests or two-way ANOVA with one repeated factor. Note that the terms “paired” and “repeated measures” have been taken by some to be different, but are the same in statistical tests [24]. The lack of a suitable protocol means the Puri and Faulkes paper could not test for the anaesthetic effects of benzocaine. The low statistical power from small sample sizes and use of inappropriate statistical tests makes it unlikely that behavioural effects of treatments could be found. Further, the use of hydrochloric acid rather than acetic acid might be a problem, because the former is much less effective than acetic acid in eliciting responses in earthworms [25]. That is, the properties of these two acids are very different. The conclusion is that the study of Puri and Faulkes (2010) [17] did not replicate that of Barr et al. (2008) [16].

### 3.2. Trade-Offs

Potential trade-offs were examined by Appel and Elwood (2009) [26] and Elwood and Appel (2009) [27]. Trade-offs should provide a method to discriminate between nociceptive reflexes, which would not support the idea of pain, and more complex behavioural decisions, which might be consistent with pain [28]. The rationale for this is that reflexes should be the same regardless of other motivational needs, whereas behavioural decisions always trade-off different needs [29]. The first such studies in decapods used hermit crabs, *Pagurus bernhardus*, which were induced to occupy shells that were adapted to enable shocks to be delivered to the abdomen. Crabs occupied either a shell that was a preferred species, or one much less preferred. Electric shocks were then applied at intervals, starting with low voltage and slowly increasing [26]. Shell species did not affect the voltage at which the crabs first showed a distinct response to the shocks by withdrawing or “shrugging”. This response appeared to be a reflex. However, crabs in the less preferred shells got out of the shells at a lower voltage than those in the preferred shells. This demonstrated that the decision to evacuate was not a reflex. Rather, it was a decision that traded-off avoiding the shock with the value of the shell. A second study also used the two types of shell, but a stable voltage of 10 shocks was delivered at 20 s intervals [27]. More crabs evacuated from the less preferred species, again showing a trade-off between avoiding the shock and retaining a high-quality shell. A third experiment by Magee and Elwood (2016) [30] asked if hermit crabs would trade-off between avoidance of shock and avoidance of potential predators. Fewer crabs evacuated from their shells if the odour of a potential predator was in the water compared to those without an odour, or an odour of a non-predator, indicating a trade-off. A fourth study by Barr and Elwood (2024) [31] used shore crabs, *C. maenas*, that normally avoid bright light by hiding in dark shelters. This experiment used shocks of 0 v, 6 v, or 12 v within the shelters, and varied the light intensity in the test arena. The key measure was to examine if the crabs would enter the shelter depending on these conditions. After fifteen trials, crabs were more likely to enter the shelter when the arena light was bright and the shock level low. This showed a trade-off between avoiding light and avoiding shocks.

A key difference between these experiments is that, with the hermit crabs, the trade-off was seen in a decision immediately following the aversive stimulus. By contrast, in the shore crab study, the animal used a memory of the shock intensity in a decision to enter the shelter, coupled with information about light intensity. In all four studies, the behaviour cannot be explained by nociceptive reflexes because it is clearly the product of decision-making. Trade-offs enable the animal to achieve a better outcome than could be achieved by reflex alone [32].

### 3.3. Long-Term Changes in Behaviour Following Noxious Stimulation, Excluding Associative Learning

Elwood and Appel (2009) [27] examined long-term behavioural changes, other than associative learning, using hermit crabs. These crabs inhabited shells that were modified so that shocks could be given to their abdomen. Some crabs were given shocks, and 20 s after the last shock, or at an equivalent time for those not shocked, the crabs were offered a new empty shell. How crabs responded to the new shell depended on whether they had been shocked or not. Shocked crabs took a shorter time to contact the new shell, spent less time assessing the shell, used fewer probes of the chelipeds in assessing the shell, and were more likely to enter the new shell. That is, being shocked within a shell increased their motivation to acquire a new shell. Because of the delay between shock and the new shell being offered, this could not be due to a reflex response. Neither could it be due to associative learning, because the animal had no previous opportunity to change shells following shock. Rather, it demonstrated an alteration of motivational state due to a noxious stimulus.

A second study extended the period between the end of the shocks and the offering of a new empty shell. Crabs were offered a new empty shell, 5 min, 30 min, 2 h, or 24 h after the shocks stopped, or an equivalent time for the non-shocked crabs [22]. There were major differences in the behaviour of the crabs when investigating shells, depending on being previously shocked or not. Overall, shocked crabs were more likely to contact the new shell, and more likely to move into the shell. They contacted the shell in a shorter time, and used few cheliped probes in assessing the new shell, and fewer shocked crabs thrust their abdomens after moving to the new shell (an activity used in post-change assessment [33]). When just those offered new shells 24 h after being shocked were examined, shocked crabs were found to have used significantly fewer cheliped probes, and were less likely to thrust their abdomen after moving to the new shell. These changes indicate that the shocked crabs no longer valued their shells as highly as did those not shocked [33]. Again, the changes in behaviour could not be attributed to nociceptive reflexes because they occurred long after the cessation of the stimulus. Rather, they were consistent with the idea that these animals experienced pain [22].

Two studies by Fosset et al. (2014, 2015) [34,35] are notable, even though they did not explicitly mention pain. These examined crayfish, some of which had been repeatedly shocked, causing escape tail flicks. They were then placed in a cross maze that had two arms in bright light and two in the dark. How the animals behaved was altered by the shocks, with shocked crayfish showing a greater avoidance of the light arms than those that were not shocked. This was described as demonstrating anxiety, which is an increased aversion to risk. It is consistent with the idea of pain being induced by the shocks, but the authors referred to stress being induced. The aversion to risk is expected to enhance the fitness of animal in times of danger, and this has been shown in studies on amphipod crustaceans [36] and squid [37], again without reference to learning.

### 3.4. Opioid Analgesia

Attention then turned to exogenous opioids, which reduce responses to noxious, potentially painful, stimuli in fish [38], amphibians [39], birds [40], and mammals [41], and opioid analgesia was suggested as a criterion for pain by Bateson (1991) [42]. In the mantis shrimp, *Squilla mantis*, and the crab, *Chasmagnathus granulatus*, responses to electrical shocks were reduced by dose-related morphine injection [43,44], and this was reversed by the opioid antagonist, naloxone, suggesting an analgesic action [44]. These findings formed a central part of reviews, suggesting that crustaceans might experience pain [13,15]. However, morphine injection was also found to reduce an escape response to a visual stimulus in the crab *C. granulatus* [45]. This opened the possibility that morphine has effects on the responsiveness of crustaceans other than a specific analgesic action. To test this possibility, an experiment was devised by Barr and Elwood (2011) [46] such that, if morphine had an analgesic effect, then a higher level of response would be seen. This involved shore crabs being placed in a brightly lit arena from which they could enter a dark shelter to avoid the light. On entering the shelter, however, some crabs received a shock, so on subsequent trials shocked crabs might be expected to avoid the shelter. Some crabs were pretreated with morphine, and it was expected that an analgesic effect of morphine would make them more likely to enter the shelter because they would not feel the shock. However, the data did not show an analgesic effect. Rather, morphine-treated crabs seemed unable or unwilling to walk and, thus, did not enter the shelter, and that was not affected by whether the shelter was paired with shock or not. After about 10 trials, the effects of morphine declined, and crabs then entered the shelter. Overall, during the second half of the 20-trial procedure, crabs that received shocks had a longer latency to enter, and were more likely to get out of the shelter following the shock, but there was no effect of morphine on these decisions [46].

A second study of the effects of morphine on the behaviour of shore crabs by Barr and Elwood (2024) used a different approach [21]. Animals were injected either with water or morphine and observed for 5 min. They were then injected in a fifth walking leg with either water or different concentrations of acetic acid and observed for another 5 min. In the first 5 min, crabs given morphine reduced the amount of time that they pressed against the sides of the observation arena, but increased the time spent rubbing and picking their mouth parts and were more likely to show a defensive display than those given water. During the second observation period, crabs given acetic acid rubbed the injected leg with other legs, were more likely to hold the injected leg off the substrate, and were more likely to autotomise the injected leg, especially with higher concentrations of acetic acid [21]. However, whist these responses are consistent with the idea of pain, morphine did not influence their occurrence. That is, there was no evidence of an analgesic effect of morphine [21]. Nevertheless, morphine did affect the behaviour of the crabs, especially reducing locomotion, and that seems to be the reason for the early, mistaken claims of an analgesic effect.

Because opioids have been suggested as a marker for pain [42], the negative findings not only fail to support the idea of pain in these animals, but they might lessen the case for pain. A weakening of the case, however, rests on a presumption that all animals that experience pain will have a modulatory system based on opioids [47]. It might be possible, however, for animals to experience pain-like states and modulate those states by non-opioid means. For example, responses to thermal stimuli were reduced by steroids in the snail, *Cepea nemoralis* [48], and other studies reported both opioid and non-opioids that moderate responsiveness to noxious stimuli [49,50,51]. Barr and Elwood (2011) [46] concluded that the criteria for pain should not include physiological mechanisms that might be taxon-specific. However, there is no doubt that, had opioids moderated responses in crustaceans in a way that is consistent with an analgesic, it would have been claimed as support for the idea of pain in these animals. Future studies should investigate if crustacean responses to noxious stimuli are modulated by other mechanisms.

### 3.5. Avoidance Learning

A key expectation of pain is that it will reduce future tissue damage and, thus, should enhance learning to avoid the situation that gave rise to the pain [52]. With pain, we expect to see rapid avoidance because that provides the best outcome in terms of reducing future tissue damage. This is because pain should include an awareness of the negative emotional state, and this is expected to direct attention to the stimuli associated with pain, leading to the rapid avoidance. Indeed, if we see learning that takes many trials, that would be good reason to suspect pain is not involved.

Magee and Elwood (2013) [52] examined avoidance learning using shore crabs, *C. maenas*, that were placed in a brightly lit arena from which they could choose between two dark shelters located at opposite ends of the tank. This species shows strong avoidance of light, and naturally uses dark shelters under rocks to avoid visual predators. Crabs were randomly selected to receive a shock when they entered a shelter and received further shocks if they remained in that shelter. Other crabs received no shocks in the first trial, but on later trials (10 trials), they were shocked if they went to the alternative shelter. Those that were shocked in the first shelter were not shocked if they subsequently went to the alternative shelter. Crabs could leave a shelter during the trial, and could go to the alternative shelter during each 2-minute trial. At the end of each trial, the crabs were removed from the arena for a short period, and then replaced in the centre of the light area. Regardless of being shocked or not in the first trial, crabs in the second trial showed a strong preference to return to the previously chosen shelter. In the third trial, however, those that had been shocked in the second trial were significantly more likely to switch their choice compared to those not shocked. That is, crabs avoided the place that resulted in a shock following two experiences in that shelter and, thereafter, tended not to go back to that shock shelter. To achieve this, they had to overcome their initial preference. Other behavioural changes were noted that reduced the number of shocks the crabs received. Crabs were less likely to enter either shelter following a shock experience rather than a non-shock experience. Further, crabs that left a shelter only did so following a shock experience, and the likelihood of leaving shock shelter increased over the ten trials. Having left a shelter, crabs often entered a shelter again in that trial, but they typically crossed the light area to enter the alternative non-shock shelter, rather than returning to the one associated with shock. Following ten trials, the position of the crab was changed, and visual stimuli over each shelter were switched for some crabs. This demonstrated that crabs had learned the direction of walking for the discrimination rather than learning visual cues.

The study by Magee and Elwood (2013) [52] replicated the situation on the shore, where crabs might sample more than one shelter under rocks and take the most suitable; however, it is not a common procedure in formal learning studies. For this reason, a second experiment was conducted. This involved placing a barrier in the test arena so that only one shelter could be accessed on each trial, and the crab was placed alternately, from trial to trial, to the side in which shock, and then no shock, would follow shelter entry [53]. The crabs were subsequently tested with the barrier removed, but no preference for the non-shock shelter was found. Thus, discrimination occurred only when both choices were present simultaneously [52] compared to sequential presentation [53], as has been found in other discrimination learning studies [54]. However, despite the lack of discrimination learning, other aspects of behaviour changed that reduced the number of shocks. Some crabs exited from the shock shelter, and the proportion doing so increased over the trials. Further, crabs got out of the shock shelter more quickly in later trials. Crabs thus switched to rapid escape as a means of reducing shocks. Crabs were also slower to enter the shelter following a shock trial compared to following a non-shock trial. That is, even without the discrimination, behaviour was modified to reduce the number of shocks.

Other studies have examined avoidance learning, but without reference to the possibility of pain. For example, Denti et al. (1988) [55] used the crab *C. granulatus* in a double-chambered device that had a dark compartment and a light compartment. Each crab was placed in the dark compartment and allowed to move into the light compartment. Some received a shock in the light compartment. Experimental animals and control animals were then allowed to go back to the dark compartment. The process was repeated and, with intervals of up to 3 h, those that had been shocked showed a longer latency than controls to enter the light compartment. A single trial was sufficient for the delay, but it was not found with a 24-hour interval. Avoidance learning has also been shown in the marbled crayfish (*Procambarus virginalis*). In a T-maze they initially showed a preference for an arm with blue light compared to an arm with white light. However, if the blue light arm resulted in electric shock, the preference was switched. One shock was sufficient in the short-term, and three exposures resulted in avoidance that lasted for 48 h [56]. Thus, there are several studies that show very rapid avoidance learning, and are thus consistent with the idea of pain in these animals.

### 3.6. Autotomy

Autotomy in crustaceans typically involves casting off a walking leg or cheliped. The break occurs at the joint between the coxa and the basis, where the limb joins the body. It is caused by muscles normally used in locomotion, and the fracture plane heals rapidly [57,58]. The muscles are controlled by motor neurons, and autotomy is a behavioural response because of a central decision [58]. Autotomy varies with other motivations, and the decision to autotomize may include information about costs and benefits of losing the limb [59].

Autotomy sometimes occurs when the limb is held [60], and this seems unlikely to involve pain. However, it also occurs when various noxious stimuli are applied to the appendage, such as heat [11], electric shock [46], a cut to a distal joint [4], injection of an appendage with formalin [18] or acetic acid [21], and inserting a spike into the membrane between the dactylus and merus sections of an appendage [5,61]. Sherwin (2001) [13] speculated that, in some cases, autotomy might be mediated by a pain-like state. It is often seen in association with other activities that are consistent with the idea of pain. For example, when hermit crabs are given shocks within the shells, limbs may be cast-off at about the time that we see trade-offs between shell evacuation and other motivational requirements [26], or when shore crabs are making decisions to avoid electric shocks to a leg [52]. In some studies, injection of formalin [18] or acetic acid [21] resulted in the limb being held in unusual positions, and use in locomotion was reduced but rubbing by adjacent limbs increased before autotomy. These activities are consistent with pain, so it is a possibility that the autotomy seen at that time is mediated by pain.

### 3.7. Physiological Stress Responses Following Noxious Stimuli

In humans, pain can lead to physiological stress. In particular, the HPA axis may be stimulated causing release of corticosteroids [62]. Crustaceans also show physiological responses to various stressors, but the main hormone is crustacean hyperglycaemic hormone (CHH) [63]. This has similar effects to corticosteroid; it converts glycogen to glucose, and lactate is also produced [4]. CHH is difficult to assay compared to glucose and lactate, and the latter products have been used as proxies for crustacean stress responses. Conneely and Coates (2024) [64] used a meta-analysis, and found that lactate was a good indicator of the welfare status of crustaceans. For example, lactate increased one minute after a cheliped was pulled off in the manner sometimes used to collect the claws of crabs, and was higher when such declawed crabs were kept with an intact conspecific [4].

CHH increases when serotonin increases, and serotonin is linked to the “*sustained apprehension of the environment*” called anxiety [65]. Anxiety was examined by Fossat et al. (2014, 2015) [34,35] by exposing some crayfish to electric fields that induced tail flicking escape responses, and flicking increased with the amplitude of the electric fields. As noted above, the electric fields subsequently increased anxiety. The shocks also caused physiological changes, including increased serotonin, which increases CHH release. Injection of serotonin without associated shocks could also increase anxiety, and injection of anxiolytic chlordiazepoxide reduced the behavioural signs of anxiety, but did not reduce serotonin [34,35]. The authors referred to the electric fields as a means of inducing stress, and did not refer to pain. Nevertheless, there are clear similarities between these studies on crayfish and those that used electric shock to induce long-term changes in behaviour in other decapods.

However, there is a problem in interpreting the results of studies that use an aversive stimulus. Because these stimuli typically induce an immediate, vigorous change in behaviour, such as the tail flicking escape responses seen in the crayfish, we cannot be certain that the physiological responses are directly caused by the stressor. They might be caused, instead, by the vigorous behaviour. Elwood and Adams (2015) [66] attempted to disentangle these possibilities by giving electric shocks to some shore crabs, but not to others. The behavioural responses differed between the two groups, with controls only showing either no movement or walking, whereas shocked crabs showed either walking or the more extreme threat responses or attempting to climb out of the observation arena. When just those that walked were analysed, it was found that those that received shock had substantially higher levels of lactate than the non-shocked controls. That is, the stress response was due to the shock, and not because of the behavioural change. These data are thus consistent with the idea that electric shock induces a pain-like state that is stressful.

### 3.8. Nociceptors

The perception of noxious stimuli typically involves the activation of nociceptors. The impulses are conducted along nerve fibres and vertebrates have two types, some being myelinated and others unmyelinated. The fast myelinated fibres are used in reflex responses, and the slower unmyelinated fibres conduct to the brain to initiate pain responses. Those in invertebrates are not myelinated [67] and like those involved in pain in vertebrates, but this tells us little about the possibility of pain. However, because pain experience, due to tissue damage, depends on nociception, a lack of nociceptors would suggest that the animal was insensitive to noxious stimuli. While the presence of nociceptors per se does not demonstrate that pain is experienced, it would add to the possibility of pain [38].

Puri and Faulkes (2010) [17] isolated antennules from crayfish, *Procambarus clarkia*, and recorded electrical activity in the exposed nerve when the distal tip of the antennule was brushed with HCl or NaOH, but they found no consistent nerve responses. That is, they found no evidence for nociceptors responding to acid and alkaline chemical stimuli. The same authors used a similar approach to test responses to capsaicin or isothiocyanate or high and low temperatures [68]. The neurons in the antenna did not change their firing rate following application of capsaicin or isothiocyanate, and there was no behavioural aversion to those substances. There were no responses to low temperature, but high temperature (60 °C) caused a number of activities that included escape and attempting to hold the shaft of a hot probe (soldering iron) away from the animal. In addition, there was clear evidence of neuronal responses to heat in the isolated antennule that was consistent with the idea of nociception and, thus, the presence of nociceptors [68].

Kasiouras et al. (2024) [69] recently investigated possible nociceptive responses to mechanical and noxious chemical stimuli in shore crabs, *C. maenas*. These were assessed by extracellular multi-unit electrophysiological recordings from both the anterior ganglion and the circumoesophageal ganglia. Mechanical stimuli elicited shorter, more intense neural activity compared with acetic acid, and there were higher thresholds for mechanical stimuli on the claws than for other areas. Acetic acid triggered neural responses from all areas to which it was applied; however, the antennae and antennules did not respond to mechanical stimuli. This latter observation matches the low behavioural responses to mechanical stimuli applied to the antennae noted by Barr et al. (2008) [16]. In general, however, the results suggest not only the presence of nociceptors, but also that different neuronal responses were caused by different stimuli, both within and between modalities [69].

The conclusion is that these different studies on the behaviour and neural responses indicate a sensitivity to noxious stimuli, and the data are consistent with the idea of nociceptors in crustaceans. Although the specific nociceptors were not identified, the study was clear in showing that some receptors were stimulated by tactile and chemical stimuli and these, in turn, stimulated components in the CNS. These CNS components would be required for the control of behavioural responses noted in other studies [16,20,31]. Further, because the pain experiences are generated in the brain, the demonstration of electrical changes in the anterior ganglion (brain) in the head of the crab agrees with a key expectation of pain.

## 4. Objections to the Idea of Pain in Crustaceans

The possibility of pain in crustaceans has been rejected by a substantial group of authors, many with links to the fishing industry (e.g., Rose 2014; Stevens et al., 2015; Diggles 2019; Diggles et al., 2024) [70,71,72,73]. One claim is that crustaceans respond to noxious stimuli purely by reflex [70]. However, experimental work by others has shown that many responses cannot be explained by reflexes [1,28,74]. Another objection suggests that any animal lacking the brain areas involved in human pain would be unable to experience pain [70,75]. This contention was based on “*the bioengineering principle that structure determines function*” [75], and that only one structure can have a particular function. However, morphologically different brains can have similar functions, as happens for vision in cephalopods, humans, and crustaceans. Thus, dissimilar morphology cannot be used as a valid argument to reject possible pain [1].

It is further claimed that the brains of crustaceans are too small for the necessary neural computation. However, the brains of crabs and lobsters are likely to be larger than those of many vertebrates. Broom (2007) [15] and Elwood et al. (2009) [76] noted that brain size does not necessarily equate to complexity of function, as shown by the cognitive abilities of bees [77]. The brains of decapods are surprisingly complex [78], with different areas showing clear functional separation, and they enable complex behaviour [33]. It is not known how many neurons are required to experience negative affective states, but it might not be particularly large [32]. Crump et al. (2022) [79] provide a good review of the brain structure of decapod crustaceans, and conclude that they have many of the requirements to make pain a possibility.

To cast doubt on the possibility of pain in fish and crustaceans, there have been attempts to discredit the scientific integrity of those involved in experimentation. For example, Rose et al. (2014) [70], stated that the experimental work has not used the “*detached tradition expected of basic science*” and has been “*mission oriented*”. Rose et al. (2014) [70] further claimed that it is often “*faith-based research*” and that “*these biases have an insidious impact on the credibility of the “science” surrounding aquatic animal welfare*”. These comments were refuted by Elwood (2021) [74] because they did not match reality. Diggles (2019) [72] suggested that “*scientific claims that fish or crustaceans “may feel pain” have been largely based on a few dubious and disputed studies done on a small number of animals and species*”. This was also refuted by Elwood (2021) [74], who noted that the thirteen experiments from his laboratory, published by that time, used four species with an average sample size of 91.7 (range 40–244). These studies were published in international, peer-reviewed journals, and none of the numerous reviewers found the sample sizes small. Further, Diggles et al. (2024) [73] claim that “*Purportedly authoritative and comprehensive reviews that “cherry-pick” literature to support their narrative fall well short of the scientific rigor that is needed to underpin policy decisions*”. The article goes on to suggest that the responses to acetic acid and sodium hydroxide found by Barr et al. (2008) [16] had not been found by Puri and Faulkes (2010) [17]. It is noted above that the design and analyses of the experiments were very different, so the later one cannot be regarded as a replication of the earlier. Further, Diggles et al. (2024) [73] failed to mention the study by Elwood et al. (2017) [19] that demonstrated responses of shore crabs to acetic acid, and did not mention the study by Dyuizen et al. (2012) [18] that reported prolonged responses to formalin injected into the claws of crabs. Thus, the claim by Diggles et al. (2024) [73] that the Barr et al. (2008) [16] study had not been replicated is misleading. It is based on cherry-picking, about which they previously complained. Since those assertions about a lack of replication, three more studies have demonstrated responses to acids and/or bases [20,21,69]. Thus, we now have six studies on decapods that demonstrate responses to chemicals that are noxious to mammals.

Diggles et al. (2024) [73] suggest that responses to noxious stimuli by crustaceans (and fish) are too readily interpreted as being “*consistent with pain*”. However, there are examples of nociceptive reflexes being offered as an explanation when that is appropriate [16,20,26]. For example, when glass prawns had noxious chemicals applied to an antenna, they often showed a tail flick escape response that was described as a reflex response. The reflexes simply demonstrate that the noxious chemicals are perceived by animals [16,20]. Similarly, when hermit crabs were given shocks of gradually increasing intensity, they first responded by withdrawing or shrugging (crabs bend their body towards the ground with retracted appendages). Again, this was described as a reflex, and no support for pain was claimed for that response [26]. That is, the authors of those experimental studies were careful not to ascribe immediate, seemingly invariant, responses as being mediated by pain. Only complex responses, particularly those occurring a long time after the cessation of the stimulus or that involved decision-making, were thought to be consistent with the idea of pain [28,74].

Those who have conducted the scientific investigations have repeatedly acknowledged that proving pain is not possible [28,74]. However, Rose et al. (2014) [70], Diggles (2019) [72], and Stevens et al. (2015) [71] mislead their readers by repeatedly stating that the researchers claimed they had proved pain (see Elwood (2022) for examples) [74]. More recently, Diggles et al. (2024) [73] stated that review papers by Passantino et al. (2021) [9] and Conte et al., 2021) [2] concluded that “*crustaceans are sentient and feel pain*”. Again, this is not the case. For example, Passantino et al. (2021) [9] wrote: “*We accept that it is not possible to prove beyond all doubt that any animal species has the capacity to experience pain*”. Conte et al. (2021) [2] wrote that “*being consistent with pain is not the same as proving pain, and that is not possible for any animal taxon*”. These two reviews avoid the unequivocal acceptance of pain that Diggles et al. (2024) [73] claim of those papers. By contrast, although Key (2016) [75] reasonably claimed that we cannot prove pain in fish, he also stated that “*we do not have the tools at present to definitively “prove” with a single experimental approach that fish do not feel pain*”, and presumably the same applies to crustaceans. Thus, if pain in these taxa can be neither proven nor disproven then, logically, we must accept there is some possibility of pain [80].

Diggles et al. (2024) [73] go on to suggest that the term “*pain*” should not be used unless sentience can be unequivocally proved. It should be clear, however, that there can be no proof of sentience in any animal. However, the lack of total proof of sentience should not be an excuse to shut down investigations into possible pain, and it should not be an excuse for failing to provide good welfare standards to crustaceans [1,81,82]. Diggles et al. (2024) [73] then compare scientific investigations on possible pain with a belief in the tooth fairy, and conclude by invoking Winston Churchill: “*Never before in the field of human food security, has so much been put at risk for so many, based on so few verifiable facts*”.

## 5. What Indicators Should Be Used?

Over the years, different sets of criteria or markers for pain have been proposed. Some were aimed mainly towards vertebrates [42,83], some were created primarily with invertebrates in mind [1,79,82], and others attempted to cover all animals [84]. These sets differ with respect to which criteria are included, and there is considerable disagreement about which criteria are useful and/or preferred over others [79,85,86,87]. One suggestion to guide selection was to consider the expected functions of pain [88]. Because nociceptive reflexes are probably evolutionarily early, we should ask what extra pain provides [28,88]. To be considered as being consistent with pain, the responses should be different from nociceptive escape or withdrawal reflexes [28], because the latter only provide immediate short-term cessation of tissue damage. By contrast, pain is expected to reduce future damage and facilitate healing and recovery [84]. Elwood (2023) [88] used these expectations to focus on behaviour that appeared to produce these beneficial effects. He suggested that activities directed towards the specific site of damage and guarding the damage to avoid further injury would be good indicators of possible pain. The guarding might involve physical covering of a wound with other body parts or changed use of the wounded part of the body. Autotomy may also be a beneficial response to severe damage and may be an indicator of pain [21]. Pain should also enhance the ability of the animal to lessen subsequent exposure to the noxious, tissue-damaging stimuli. This might involve swift learning to avoid the situation that led to the pain and/or a reduced willingness to take risks [28,84]. The latter might be termed anxiety, shown by a heightened responsiveness to some stimuli coupled with risk aversion. This should enhance fitness, because wounded animals are often selectively targeted by predators [37,89]. Anxiety states, however, are likely to change access to, or use of, resources if there are associated risks. While the animal might give up important resources, that should be balanced against the benefit of avoiding the pain [26,27,30,31,32]. That is, we expect pain to influence behaviour in ways that promote subsequent survival [1,42,84,90].

However, other criteria have been proposed, including physiological changes, identifying brain structures that might mediate the behaviour change, the presence of nociceptors, and the descending control of nociceptive input [79,84]. Elwood (2023) [88] noted that these are secondary to the behavioural changes because they provide the mechanisms for the behavioural response, although physiological changes may promote recovery. However, some authors have argued strongly for including at least some these aspects in sets of criteria [17,68,69,79,84], and they are included in one guise or another in most sets.

Birch et al. (2021) [82] selected eight criteria in their highly influential review for the UK government, and developed a method of assessing the confidence with which each had been fulfilled (also see Crump et al., 2022) [79]. They proposed that this set should be used as a “framework” when evaluating evidence on other taxa, for example gastropods or spiders [79], and it has been used to evaluate the possibility of pain in insects [91]. However, it is not universally accepted that all eight criteria are required or have equal validity, or should be given equal weighting in the scores [86,87]. It has also been criticised because it excludes long-term changes in behaviour that are not easily explained by associative learning [85], e.g., long-term increases in the motivation of hermit crabs to obtain a new shell following shock in their existing shell [22,27] and increased anxiety following shock in crayfish [34,35].

Birch et al., 2021 [82] and Sneddon et al., 2014 [84] included self-administration of analgesics, which has yet to be investigated in crustaceans. It has been shown in other taxa, e.g., cephalopods [92] and birds [93], and has been influential in discussions about pain in those taxa. Experiments may take different forms. One is to feed recognisably different food stuffs, one of which contains the analgesic, to animals in chronic pain to see if a preference is formed for the analgesic [93]. That requires the food to be taken separately so the analgesic has sufficient time to take effect before the other is ingested. If both foods are consumed before that effect, the animal would not be able to associate one food with the analgesia. Alternatively, the analgesic or local anaesthetic could be provided in one location, and a preference for that location might indicate a preference for the analgesic or local anaesthetic [92]. Again, there are difficulties to overcome; for example, the administration of the drug should not elicit any distress, otherwise it might cause the animal to associate the location with that negative state. In both types, we need to be certain of the effectiveness of the drug. For example, had morphine been given to a crab in one location, a preference might have been incorrectly assumed if the morphine reduced walking [46], causing the animal to spend longer in that location. We would need to look for preferences shown by active choice to enter a location, not simply staying in the location. A procedure for self-administering amphetamines in crayfish (*Orconectes rusticus*) might offer a suitable technique for this type of experiment [94].

There has also been concern that the framework proposed by Birch et al. (2021) [82] might inhibit research into new criteria [85]. To promote further studies, Elwood (2023) [88] suggested three areas that might provide new insights into the possibility of pain. First, fights for resources are often violent [95], and injuries might induce pain that mediates fight decisions [33]. Thus, studies on aggression may prove useful in understanding negative affective states [96]. Second, observations of males causing internal tissue damage during mating, which reduces the probability of the females subsequently mating with another male, and of females with spines that deter males from prolonged copulations, might also be of interest [88]. Third, toxins might induce pain to deter predators. For example, Karplus (2024) [97] reviewed associations between hermit crabs and sea anemones. Hermit crabs are selective in the species of anemones they have on their shells, and may compete to obtain them. The hermits do not appear to receive stings from these anemones, but the anemones do appear to sting and deter shell-crushing predatory crabs. Hermit crabs with anemones were frequently dropped by these predatory crabs, but this was rarely seen with hermits without anemones. Further, hermit crabs with anemones showed superior survival compared to those without anemones. Such observations might open a means of assessing how the anemone stings stop the predatory crabs from attacking, and if pain-like responses might be involved.

## 6. Studies Aimed to Improve Crustacean Welfare

### 6.1. Analgesics and Anaesthesia for Crustaceans

Various studies have attempted to determine if analgesics and anaesthetics reduce possible pain or stress reactions to damaging treatments (reviewed by de Souza Valente 2022; Rotllant et al., 2023) [98,99]. For example, eye-stalk ablation in shrimp brood stock, *Penaeus* (*Litopenaeus*) *vannamei*, has been used to increase egg production in aquaculture, but there was concern that this might cause stress to the animals [7]. This was the rationale for an investigation into “*possible methods for alleviating stress due to the pain encountered by the event of eyestalk ablation*” by Taylor et al. (2004) [7]. Some animals were treated with the local anaesthetic xylocaine (also called lidocaine) prior to cutting the eyestalk and expelling the contents, or having the eyestalk pinched to squeeze out the contents. Those without the xylocaine showed an immediate tail-flicking escape response to ablation, and prolonged erratic swimming that included bumping into the walls of the tank, and took longer to resume feeding compared those with xylocaine. Curiously, no analyses were provided in the article to test for statistical significance, so the claims about their continuous measures, such as time taken to start feeding, cannot be verified. However, the raw numbers are provided for non-continuous measures, allowing for contingency tests to be performed. For example, erratic swimming was seen in 22 of the 30 not treated with xylocaine, compared to 1 out of 30 with xylocaine, Chi^2^ = 28.2 *p* < 0.0001 with Yates correction. The authors concluded that the use of the xylocaine alleviates “*stress*” due to eyestalk ablation. They now use local anaesthetic in a standard protocol in the laboratory [7]. Diarte-Plata (2012) obtained broadly similar results when they examined the effects of xylocaine following ablation or ligation of eyestalks in the freshwater prawn, *Macrobrachium americanum* [100]. Again, there was a presumption of pain in these animals, and the conclusion was that xylocaine minimised the “trauma” induced by ablation [100]. The aim in these studies was not to investigate the possibility of pain, as pain/stress was taken; nevertheless, they are similar in the broad approach to Barr et al. (2008) [16], who noted a reduction in responses to noxious chemicals applied to the antennae of glass prawns following benzocaine application.

The reviews by de Sousa Valente (2022) [99] and Rotllant et al. (2023) [98] provide thorough guidance as to the mode of administration and different uses of a wide variety of substances that reversibly reduce trauma, either by localised administration or by general anaesthesia, and are likely to be used by researchers or those in aquaculture settings. The aim of these studies is to improve the welfare of these animals.

### 6.2. Studies on the Effects of Declawing Crabs

In some crab fisheries, the claws are removed and the live animal thrown back to the sea. This is said to be a humane method because crabs are known to shed claws (autotomy), and is said to conserve stock because crabs might survive, regrow missing claws, and reproduce [4,5,61]. However, it has been demonstrated that claw removal of European brown crabs, *C. pagurus*, typically by pulling and twisting, results in a high death rate due to tissue damage and haemolymph loss that is not seen in those induced to autotomise their claw [4]. Should they survive the immediate impact of losing one or both claws, they will face reduced feeding ability because they are unable to crush bivalves or other prey with a hard exterior [101]. Thus, starvation is a possibility. The reduced food intake also means that regeneration is less likely. Regeneration is also restricted to animals that are growing and moulting, and large crabs with large claws may not moult and do not regenerate. For these reasons, claw removal has been investigated to assess the impact of the “harvesting” methods.

The UK Crab Claws (Prohibition of Landing) Order 1986 [102] prohibited landing claws that had been detached from live edible crabs, *Cancer pagurus*, but this was revoked in Scotland, England, and N. Ireland in 2000, and by Wales in 2001 [4]. In *C. pagurus*, both claws may be removed and the live animal returned to the sea [103]. Patterson et al. (2007) [4] found that removing just one claw by twisting and pulling caused a substantial stress response, as shown by increased lactate and glucose in the haemolymph, but this was not seen when crabs were induced to autotomise. Mortality was higher in declawed crabs compared to those induced to autotomise, and declawed crabs had larger wounds compared to those that were autotomised. Further, crabs that died after de-clawing had larger wounds than those that survived, indicating that death was due to the wound. The loss of a claw by induced autotomy did not appear to reduce the motivation to feed, but did affect the ability to feed on intact mussels [101]. McCambridge et al. (2016) [23] found that claw loss reduced the competitive ability of these crabs in fights for resources, but those that had the claws forcibly removed fared worse than those induced to autotomise.

Duermit et al. (2015) [104] found that claw removal in stone crabs, *Menippe mercenaria*, induced low mortality that was related to the size of the wound. However, claw removal restricted the diet of the crabs because they could not feed on bivalves. No stone crab regenerated a legal-sized claw following the first moult after claw removal, and no large crab moulted within 11 months. They concluded that crabs with claws removed were unlikely to provide legal-sized claws again. Further, Walus et al. (2023) [5] reported that survival of stone crabs was greater when claws were removed by induced autotomy rather than by forced breaking, and was greater if one rather than two claws were removed. These findings were confirmed in the red rock crab, *Cancer productus*, fishery [61]. These and other studies are shedding light on the negative impacts of claw removal fisheries and might point the way to enhance conservation and welfare.

### 6.3. Humane Killing

There have been many studies that aimed to reduce presumed pain, stress, or trauma that might occur when crustaceans are killed, for example, when boiled or butchered while alive (reviewed by Conte et al., 2021) [2]. A key advance for humane killing came with the development of the “CrustaStun”, a device that electrically stuns and kills crabs and lobsters. This involved a collaboration between Charlotte and Simon Buckhaven (both barristers), who conceived the idea, and Bristol University, who developed the device in 2009. The “CrustaStun” is a metal box containing a tray with a wet sponge and an electrode. When the lid is closed, the wet sponge contacts the animal and conducts a current that immediately disrupts the neural activity [105]. The efficacy of this stunning was investigated by Neil et al. (2022) [106] using central and peripheral neural recordings as measures of the state of sensibility. They also examined whether electrical stunning acts as a physiological stressor, by assessing haemolymph L-lactate. Evidence of such stress would indicate a slow and less humane effect of the CrustaStun. Neural activity within the CNS and in the peripheral nerves, however, ceased on stunning, indicating a loss of sensibility, and the animals became unresponsive to external stimuli. Some animals might recover from short periods of stunning, but a ten second stun was sufficient to kill. However, neural activity is disrupted almost immediately, and there was no increased L-lactate following stunning, suggesting the animals were not stressed by the process [106,107,108].

The “CrustaStun” was developed for use in restaurants where single animals are stunned, killed, and cooked as required. However, it is not suitable for processing large numbers of animals. To fill this demand, a dry electrical stunning system with 10 tons per hour capacity was developed by Roth and Grimsbø [107] for processing crabs. Roth and Øines [108] compared electrical stunning with other slaughter methods for edible crabs, *C. pagurus*, and confirmed that electricity rendered the crabs insensible (unresponsive to stimuli) within 1 s, and this was the most efficient stunning method for that species. These researchers recommended that electrical stunning should be employed before cooking or carving these crustaceans [108]. However, they also suggested that piercing to destroy the anterior ganglia was an effective method of killing the animal, if performed by a trained person.

The potential of electrical stunning is being explored for species other than edible crabs and lobsters, for example, with whiteleg shrimp, *Penaeus vannamei* [109]. However, it has not yet been developed for very small shrimp and prawns. These are typically killed by chilling, suffocation, or boiling [2], but these methods may have different welfare impacts compared to larger animals. For example, smaller decapods undoubtedly would die more quickly than large ones in boiling water [2]. This was investigated by Lauridsen et al. (2024) [110] by boiling previously killed decapods of different species, and hence shapes and sizes, and recording internal temperature. With a starting temperature of 10 °C, edible crabs, *C. pagurus*, took 161 s for the anterior ganglia to reach the presumed “stunning” temperature of 26 °C, whereas small species such as the Baltic prawn, *Palaemon adspersus*, took 8.8 s. Given the very different times to presumed stunning, it was suggested that this should be considered by those responsible for animal welfare legislation. They suggested that boiling might be the most humane method of killing the very large numbers of these very small decapods [110]. No doubt there will be different views on that suggestion, but at least there are moves to explore methods, other than electrical stunning, that might minimise suffering.

### 6.4. Husbandry Improvements in Aquaculture

Most research into methods used in prawn/shrimp aquaculture is geared towards improving productivity. However, that may also be relevant to the welfare of the animals, because those that are healthy, growing well, and have low mortality are also likely to have good welfare [111]. This has received attention from the group “Rethink Priorities”, and the review by McKay et al. (2023) [112] provides a carefully considered, comprehensive review of the problems that might impact farmed prawn/shrimp welfare (see also Albalat et al., 2022) [113]. Farmed shrimp primarily comprise three species that differ in requirements [3]. *Penaeus vannamei* and *Penaeus monodon* are marine, whereas *Macrobrachium rosenbergii* is freshwater; however, these species require different salinities at different stages of the life cycle [112]. Their food requirements change as the animals grow from larval to juvenile or adult stage. The food provided, or otherwise available, is likely to impact welfare, but the industry is driven by cost considerations that influence the type and amount of food, and frequency of feeding. In practice, the animals may not receive their preferred foods, and may not receive feeds that are regularly timed. The cost effectiveness of supplying food may conflict with good welfare; for example, reduced feeding of *P. monodon* leads to increased cannibalism [114] and increased aggression in *M. rosenbergeii* [115]. Environmental conditions vary in terms of oxygen, temperature, salinity, pH, ammonia pollutants, and stocking density, which all impact the well-being of the animals. For example, high stocking levels leads to some animals being excluded from resources due to aggressive competition [115]. Various diseases are problems for health, productivity, and welfare, and farms are keen to avoid infections [112,113]. Further, eyestalk ablation is employed to obtain a greater predictability of spawning. One or both eyestalks are ablated, and the contents removed by pinching, slitting, and squeezing, and may involve cauterisation or ligation [112,113]. However, such techniques may negatively impact the health of the females, and their use appears to be in decline [112,113,116]. Major welfare problems may arise in the harvesting, transport, and slaughter of these animals. Slaughter is often by suffocation, and large numbers are placed in sacs without water; others are killed in ice slurry [112]. However, as noted below, some retailers are now demanding electrical stunning to slaughter these animals. The highlighting of how husbandry methods might impact welfare is directing research efforts to find methods that minimise problems. To that end, Pedrazzani et al. (2024) [117] have developed a general welfare index for farmed shrimp/prawn, *P*. *vannamei*, to measure the degree of welfare. This uses 31 indicators that offer a comprehensive assessment framework. The use of these quantitative indicators should help to improve living conditions.

## 7. Changing Attitudes

### 7.1. Charities and Associations

In recent years, several long-established organisations have campaigned or supported the idea of improving welfare for crustaceans, and others have begun work with that specific remit. People for Ethical Treatment of Animals (PETA) have campaigned against mutilation of live lobsters and crabs since no later than 2013, sometimes by using covert films of fishing or processing, and arguing for not using these animals as food [6,118,119] or, in the case of terrestrial hermit crabs, as pets [120]. The UK organisation, Crustacean Compassion, started in 2016 and has been involved in several campaigns that highlight a need for improved welfare for decapods [121]. They have campaigned against sending shrink-wrapped live crustaceans through the postal service. Subsequently, they have urged the government, retailers, and fisheries to improve their standards with respect to the way these animals are treated. In 2019, the Royal Society for the Protection from Cruelty to Animals (UK) (RSPCA) held a conference on animal sentience, and subsequently stated that they believed there is sufficient scientific evidence that decapods should be considered sentient, and should be protected appropriately by legislation. “*This would help ensure they are no longer subjected to some of the current practices, like boiling crabs and lobster alive, that cause serious pain and distress*” [122].

The British Veterinary Association produced a policy document in 2020 [123] in which they recognised that decapods are sentient, and capable of experiencing pain and distress. They supported the principle that commercially caught decapods should be stunned before slaughter. This view was supported by the Humane Slaughter Association (UK) [124], and they funded research to improve the welfare of decapods at slaughter. Other organisations that have campaigned for better welfare for crustaceans include Rethink Priorities [125], the Australian RSPCA [126], The Animal Law Foundation [127], Animal Aid (2014) [128], and the Eurogroup for Animals [129].

The New York Declaration on Animal Consciousness in 2024 was a key move to inform about current scientific evidence and to change attitudes to the welfare of these animals [130]. This stated that “*the empirical evidence indicates at least a realistic possibility of conscious experience in all vertebrates (including reptiles, amphibians, and fishes) and many invertebrates (including, at minimum, cephalopod molluscs, decapod crustaceans, and insects)*”. Further, “*when there is a realistic possibility of conscious experience in an animal, it is irresponsible to ignore that possibility in decisions affecting that animal. We should consider welfare risks and use the evidence to inform our responses to these risks*”. This wording is particularly welcome because of the balanced view in discussing a “*realistic possibility*” of pain rather than stating that there is no doubt.

### 7.2. Regulations for Crustaceans in Science

The United Kingdom regulates the use of animals in scientific investigations via the Animal (Scientific Procedures) Act (1986) [131], but the term “animals” in that Act excludes decapods. The European Union discussed protection for decapod crustaceans used in scientific investigations, and a review and recommendations were produced in 2005 [132]. This made a case for sentience in decapods, but they were not included in the 2010 European Directive on the protection of animals used for scientific purposes [133]. This was, in part, influenced by a response by the UK parliament in 2009 that supported the inclusion of cephalopods, but considered the evidence for decapod sentience inconclusive [134].

However, the situation changed in the United Kingdom when a bill concerning sentience and welfare of animals was proposed. This did not consider invertebrates in the original draft, but amendments were suggested to include cephalopods and decapods. The Department for Environment, Food & Rural Affairs commissioned a review of the scientific evidence, and concluded that it was likely that both taxonomic groups were sentient and could suffer [82]. The review by Birch et al. (2021) [82] also made recommendations that these invertebrates should be included in the Animal Scientific Procedures Act (1986) and should be protected in the aquaculture and fishing industry. The amendment was accepted in the final Animal Welfare (Sentience) Act (2022) [135] that recognised decapods and cephalopods as sentient. However, no further legislation in the United Kingdom has been passed that would provide specific protections. Nevertheless, a consultation period was initiated in 2023 [136], including a call for evidence [137] about bringing decapods into the Animal (Scientific Procedures) Act (1986), which suggests that some change is imminent. Various other jurisdictions have some level of control on the use of decapods in science [9,136]. There have been several recent reviews that offer suggestions for good husbandry methods for these animals in research settings [9,136,138,139] and how various obstacles to bringing this diverse group within legislation might be overcome, or at least noting the major problems that need to be overcome [136].

### 7.3. Regulations for Crustaceans in the Food Industry

In Switzerland, decapods are protected by the Animal Welfare Ordinance 2008, regarding animal husbandry, transport and slaughter, and in 2018, boiling as a means of killing was banned [2]. Instead, they must be electrically stunned before killing, and cannot be transported on ice. Boiling live lobsters is also banned in New Zealand and Italy [2]. Decapods are protected in Norway, France, New Zealand, and several states in Australia [9]. However, as noted above, decapods are currently excluded from UK animal welfare legislation, so that processors and retailers do not have to consider their welfare during storage, handling, or slaughter [2].

### 7.4. Fishing, Aquaculture, Transport, and Retail

Despite limited legislation in the UK, various retailers have made moves to improve the welfare of the decapods they sell. For example, Tesco and their supplier, Hilton Sea Food, are attempting to improve slaughter methods for the millions of white-leg shrimp by using industrial electrical stunning [109]. Their announcement (circa 2021) notes: “*Hilton Seafood, Tesco and Amanda Seafood engaged in commercial trials of electrical stunning of whiteleg shrimp, P. vannamei, to assess welfare benefits at slaughter. Tesco approved the process in July 2020, since which, approximately 80% of the P. vannamei supplied to Tesco through HSF have been electrically stunned*”. The stated aim is to achieve 100% of *P. vannamei* to be electrically stunned in the supply chain [109]. Similar changes are being brought in by other major UK retailers to enhance the welfare of many millions of decapods each year [140]. For example, Waitrose [141], Morrisons [142], and Marks and Spencer [143] are all claiming to be enhancing the welfare of decapods at slaughter. Retailers are also banning the sale of live decapods and the use of eyestalk ablation in aquaculture of their products. These changes by retailers and processors are being monitored by Crustacean Compassion with a view to encourage further uptake [144].

## 8. Conclusions

Discussions about crustaceans being able to experience pain have a long history [13], but they often comprised speculation based on anecdotal evidence. Experiments that specifically asked if pain might be experienced took guidance from these early debates, and from studies that probed the question for other taxa [74]. Many experiments have now provided data that are consistent with the idea of pain in crustaceans, especially in decapods. However, that does not mean that pain has been proved; rather, it opens the possibility of pain. With each study that is consistent with pain being felt, the possibility increases, but it is doubtful if we will ever know for certain if they do or do not. Not all of the data are consistent with pain because some responses are like those expected of nociceptive reflexes. However, they at least indicate that the animal responds to stimuli such as heat, acid, alkaline or electric shock.

Crustaceans do not respond to all the noxious stimuli that affect mammals. There is no evidence that they respond to cold [68] or to capsaicin [19,68]. Another difference to mammals is that morphine appears not to act as an analgesic in crustaceans [21,46], but it does induce abnormal behaviour, so there must be receptors. Curiously, it was an early suggestion of opioid analgesia, presumably because of abnormal behaviour, that was one of the reasons given for suggesting that pain might occur in this group [13,15]. Despite these few differences, most experiments have shown similarities with mammalian responses in terms of behaviour and physiology, and these studies have been referenced by many organisations that aim to improve the welfare of crustaceans, e.g., the Crustacean Compassion, Royal Society for the Prevention of Cruelty to Animals, Humane Slaughter Association, and British Veterinary Association. They also formed much of the evidence included in the review of sentience for the UK government [79,82], and thus influenced the inclusion of decapod crustaceans in the Animal Welfare (Sentience) Act 2022 of the United Kingdom [135], which recognises that these animals are sentient. By contrast, the New York Declaration [130] said that there is a possibility of sentience. However, others, e.g., Diggles et al. (2024) [73], argue vigorously that crustaceans cannot experience pain or suffer, and that no changes to legislation or practice should be made.

Despite the recognition of decapod sentience in the UK, there have been no changes to other legislation that would provide welfare enhancements. Such changes, however, are being considered. Nevertheless, it has provided a powerful impetus to the many bodies that push for improved welfare. Further, major UK retailers are requiring changes to the way the animals are slaughtered, i.e., a shift from suffocation to electro stunning of the billions of shrimp/prawns that are sold. There are also requirements for cessation of eye ablation in production systems, and a cessation in the sale of live animals. That is, there have been substantial changes in the way that these animals are viewed and used in the UK and elsewhere. However, it is impossible to calculate how many animals are being caught or produced under new guidance on welfare standards, but it is likely to be a small percentage of current world production.

## Data Availability

Not applicable. No new data in the review.
